# Linezolid toxic optic neuropathy: A case report and review of visual prognosis

**DOI:** 10.1016/j.ajoc.2023.101922

**Published:** 2023-08-26

**Authors:** Kevin J. Toolan, Jacob Fondriest, Kaitlin Keenan, Thomas Mizen, Milena Stosic

**Affiliations:** a Department of Ophthalmology, Rush University, Chicago, IL, USA; b Department of Neurological Sciences Faculty and Staff, Rush University, Chicago, IL, USA

**Keywords:** Linezolid, Toxic optic neuropathy, Optic nerve, Ocular toxicity, Non-organic visual disorders

## Abstract

**Purpose:**

To describe a case of linezolid toxic optic neuropathy in a 71-year-old female and review the relevant literature.

**Observations:**

An adult female with progressive, symmetric vision loss was hypothesized to have linezolid toxic optic neuropathy. Following cessation of linezolid, the patient experienced improvement in visual function over two months.

**Conclusions and importance:**

Patients diagnosed with linezolid toxic optic neuropathy can expect some return of visual function after cessation. The degree of return does not correlate to the cumulative dose of the drug. The goal of this study was to summarize and add to the current body of literature on the topic.

## 1 Introduction

Linezolid is a potent antibiotic frequently used as a second or third-line agent in cases of intolerance to first-line antibiotics or microbial resistance.^1^, ^2^, ^3^, ^4^, ^5^ It is active against *MRSA,* as well as *M. Tuberculosis* and administered orally. The synthetic oxazolidinone binds and blocks the 23s rRNA portion of the 50s ribosomal subunit in bacteria as well as the analogous mitochondrial 16s rRNA in humans.^3^^,^^5^ Side effects of linezolid include myelosuppression, serotonin syndrome (due to its weak action as a monoamine oxidase inhibitor), gastrointestinal upset and peripheral neuropathy. It is rarely used for extended periods of time, but there have been case reports of optic neuropathy associated with prolonged linezolid use, suspected secondary to mitochondrial dysfunction. In this report, we present a case of linezolid associated optic neuropathy, and a brief review of the natural course of this rare condition.

## 2 Case report

**Fig. 1:**
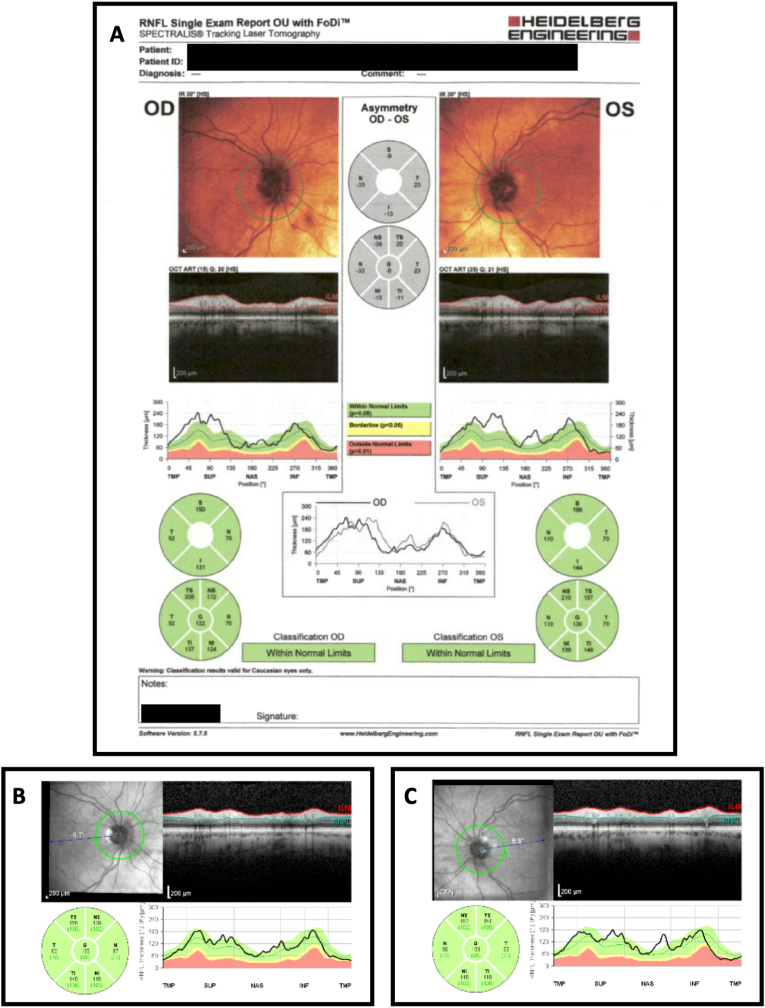
(A) OCT OU from admission. (B) OCT OD one month after presentation. (C) OCT OS one month after presentation.

**Fig. 2:**
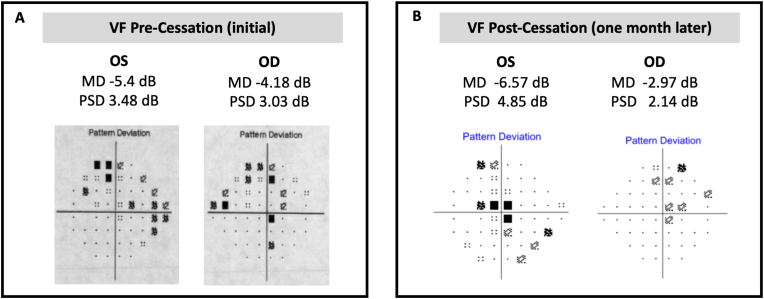
(A) Visual fields from initial presentation. (B) Visual fields one month after cessation.

A 71-year-old female with past medical history of hypertension, obesity, atrial fibrillation, and bilateral total knee arthroplasty complicated by right knee prosthetic joint infection on chronic suppressive therapy with linezolid, who was referred to neuro-ophthalmology for progressive, symmetric vision loss over three weeks. She self-described her visual defect as a blurriness like one she experiences when adjusting between bright and dim light. Her workup prior to referral returned a normal MRI brain and orbits with and without gadolinium contrast, Erythrocyte Sedimentation Rate (ESR) and C-Reactive Protein (CRP). She denied dietary restrictions/limitations though took a B12 supplement, endorsed 2–4 alcoholic drinks per month, and was a former occasional smoker 40 years prior to presentation. Best corrected visual acuity was 20/150 in both eyes, pupillary light reaction was intact and within normal limits and there was no relative afferent pupillary defect, and the patient was pseudophakic in both eyes from 1 year prior with excellent postoperative vision until these symptoms. A dilated fundus examination showed mild, symmetric elevation of the superior poles of the optic nerves with cup/disc ratios of 0.05, but no optic disc hemorrhages or obscuration of any vessels. The patient read 1/14 standard Ishihara color plates in each eye. Optical coherence tomography (OCT) of the optic nerves confirmed mild thickening of the superior retinal nerve fiber layer (Fig. 1A). Humphrey visual field testing showed a central scotoma with patchy superior depression in both eyes (Fig. 2A).

She was admitted for a neuroophthalmological workup which included a lumbar puncture, with opening pressure of 25. CSF studies, including infectious PCRs and cultures, were unrevealing (Table 1). Serum studies including nutritional deficiencies were similarly nonexplanatory. She was administered IV methylprednisolone 500mg BID for 5 days and there was no change in her symptoms or exam upon completion. She was not prescribed any additional oral or IV steroids and a temporal artery biopsy was not performed.

**Table 1:** Laboratory test and Imaging conducted during admission.

Laboratory Testing	
Test	Result
**Autoimmune**	
NMO (aquaporin-4 antibody)	Negative
**Cerebrospinal Fluid**	
Opening pressure	25
Cryptococcal antigen	Negative
Fungal culture	No growth
Lyme IgG	Negative
CMV	Negative
VZV	Negative
HSV-1	Negative
HSV-2	Negative
Syphilis	Negative
HIV	Negative
Culture	No growth
Culture (AFB)	No growth
Gram stain	No organisms
**Systemic inflammatory markers**	
ESR	14 (RR 0–27)
CRP	<5 (RR 0–8)
**Vitamins and minerals**	
Cu	93 (RR 80–158)
Zn	89 (RR 44–115)
B1	140.4 (RR 66.5–200)
B6	8.7 (RR 3.4–65.2)
B12	224 (RR 210–920)
**Metabolic**	
Homocysteine	14.93 (RR 4.44–13.56)
Methylmalonic acid	410 (RR 0–378)
**Imaging**	
MRI Orbits	Unremarkable

Her medical history was significant for several years of bilateral lower extremity polyneuropathy and interstitial cystitis. She was treated with pentosan polysulfate 25mg daily for two years, but stopped a year prior to the vision loss. Additionally, she had undergone bilateral knee arthroplasty complicated by poor wound healing requiring wound VAC and delayed closure. In the year prior to presentation, she underwent arthrocentesis for continued knee pain with purulent fluid culture positive for *Corynebacterium striatum* after incision and drainage. Due to the chronic infection with retained hardware, she was recommended lifelong bacterial suppression. She was initially treated with IV vancomycin but was transitioned to daptomycin after she developed eosinophilia. Despite this medication change, eosinophilia persisted, resulting in a final antibiotic transition to linezolid.

It was hypothesized that the patient had two significant drug exposures. Given the low dose of pentosan polysulfate, absence of RPE atrophy or macular disease, and noted optic nerve edema, pentosan polysulfate was determined to be less likely the cause of vision loss. In contrast, linezolid has been documented to present with a very comparable clinical picture to that of our patient.^6^ After consulting infectious diseases, the patient opted for linezolid cessation, and it was replaced with erythromycin. At the time of discontinuation, the patient had been taking linezolid 600mg twice a day for 12 months.

Four weeks after linezolid discontinuation, she presented with visual improvement to 20/40 -1 OD and 20/150 + 1 OS. OCT showed an reduction in retinal thickness (Fig. 1B and C) and Humphrey Visual Field testing showed a shrinking central scotoma OU (Fig. 2B). She continued to experience bilateral lower extremity neuropathy. Two months later, she reported subjective improvement in being able to see the letters on her cell phone.

## 3 Discussion

Linezolid can be useful for the treatment of drug-resistant bacteria, including MRSA, *Enterococcus* and *M. Tuberculosis*. In the FDA drug label for linezolid, optic neuropathy resulting in loss of vision is noted for patients taking the drug longer than 28 days. It notes that formulations provided for longer than 28 days have not been well studied in clinical trials. Because linezolid toxicity is rare, patients may undergo evaluation and treatment for other more common causes of optic neuropathy before the decision to discontinue the medication is made, as was the case for this patient.

We propose that linezolid toxicity is the more likely etiology for this patient's vision loss because of the classic symptoms, failure to improve with steroids, subsequent successful improvement with cessation of the medication, and otherwise negative evaluation. Though opening pressure was borderline elevated at 25 cm H2O, her presentation was not consistent with papilledema given the absence of diplopia, pulsatile tinnitus, or transient visual obscurations. Another possible etiology considered was nutritional, serum studies did identify a borderline low B12, and borderline elevated MMA and homocysteine, however the degree of B12 deficiency was insufficient to explain the severity of her symptoms, though it cannot be excluded as a contributing factor. We were unable to characterize the entire natural progression of visual improvement after discontinuing linezolid as our patient was lost to follow up. A brief review of the literature was conducted to determine visual prognosis following linezolid cessation.

Eighteen patients across twelve case reports were reviewed and data was collected on linezolid dose and length of treatment as well as time to visual recovery.^1^^,^^2^^,^^4^^,^^5^^,^^7^, ^8^, ^9^, ^10^, ^11^, ^12^, ^13^, ^14^ Recovery was typically described in case reports as an improvement in visual function to an acceptable level, usually assessed by visual acuity, visual fields, color vision or improvement in OCT-RNFL edema. It was not uncommon to have evidence of permanent damage, despite subjective return of visual function. Of the 11 patient reports that reported this finding, 64% of them had RNFL thinning or ONH pallor after subjective recovery. Other permanent sequelae after subjective recovery include one patient (13%) with decreased color vision, six (33%) with visual acuity worse than 20/30, and seven (44%) with residual visual field defects (Table 2). Of note, four of the eighteen patients in our review had also been exposed to ethambutol. All four of these patients were a part of the twelve patients that experienced continued visual defects (visual field, visual acuity, color vision and RNFL thinning or ONH pallor).

**Table 2:** Literature review of 18 patient cases of linezolid toxic optic neuropathy.

Article	Linezolid Dose (mg)	Time to Symptom Onset	Time to Recovery (after cessation)	Ethambutol Use?	Residual VF Defects?	VA worse than 20/30	Decreased color?	RNFL thinning or ONH pallor?	Disc Swelling?
Aljebreen et al.	600 BID	5 months	3 months	No	No	No	No	Yes	Yes
Chaitali and Ramchandani	600 daily	9 months	3 months	No	Yes	Yes	N/A	N/A	No
Dempsey et al.	600 BID	12 months	25 days	No	Yes	Yes	Yes	N/A	No
Javaheri et al.	170 BID	12 months	3 months	No	N/A	No	N/A	Yes	Yes
Karuppannasamy et al.	600 daily	6 months	1 months	Yes	Yes	No	No	Yes	Yes
Khadilkar et al.	300 BID	10 days	2 months	No	No	No	No	No	Yes
Khadilkar et al.	300 BID	6 months	15 days	No	No	No	No	No	No
Kiuchi et al.	600-1200 daily	25 months	5 months	No	No	No	N/A	N/A	No
Kulkarni and Del Priore	600-1200 daily	56 months	3 months	No	No	Yes	N/A	N/A	No
McKinley and Foroozan	600 daily	11 months	4 months	No	No	No	N/A	Yes	No
McKinley and Foroozan	600 daily	10 months	3 months	Yes	Yes	Yes	No	Yes	No
Libershteyn	600 BID	Months (unspecified)	3 months	Yes	Yes	Yes	No	Yes	No
Mehta et al.	600 daily	11 months	22 days	No	N/A	No	N/A	No	Yes
Mehta et al.	600 daily	11 months	45 days	Yes	Yes	No	N/A	No	Yes
Mehta et al.	600 daily	10 months	45 days	No	No	No	N/A	N/A	Yes
Mehta et al.	600 daily	7 months	78 days	No	Yes	No	N/A	N/A	Yes
Rucker et al.	600 BID	6 months	4 months	No	No	Yes	N/A	Yes	No
Rucker et al.	600 BID	5 months	3 months	No	No	No	No	N/A	Yes

**Fig. 3:**
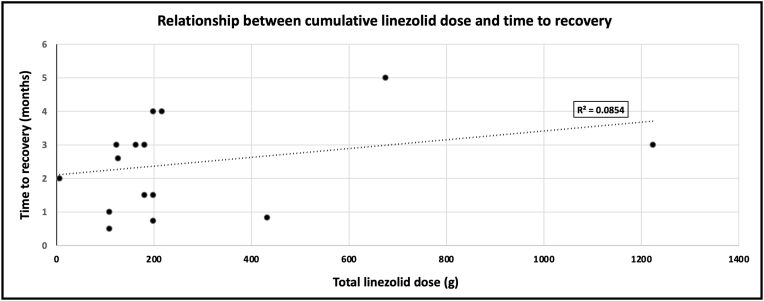
Relationship between cumulative linezolid dose and time to visual recovery after cessation. Data on seventeen patients displayed on scatterplot with best fit line.

Some studies have shown resolution of visual deficit as rapidly as fifteen days after cessation even after six months of treatment.^9^ At our patient's one month follow up, the visual acuity in her left eye remained unchanged, raising the question of correlation between cumulative dose and time to visual recovery. Using the aforementioned case reports, we compared total cumulative dose (grams) of linezolid to the time of reported visual recovery. Analysis of seventeen patients in our review showed a very weak positive correlation (R^2^ = 0.085) suggesting that no clear relationship exists between the two variables (Fig. 3). However, this data is limited and is not controlled for possible confounding variables. Given the paucity of data, physicians should use caution when giving anticipatory guidance to patients with linezolid toxicity as there is no clear correlation between their prior linezolid regimen and time to resolution of symptoms.

At the time of presentation, our patient reported bilateral peripheral neuropathy that started more than a year ago but worsened following her orthopedic procedures. While she may also have suffered from a mild B12 deficiency, the degree of visual impairment was out of proportion, suggesting that the more likely explanation of her symptoms was linezolid. When our patient was last examined after linezolid cessation, she showed signs of improving optic neuropathy with persistent peripheral neuropathy – a finding consistent with other studies.^3^^,^^15^ The order of presentation of peripheral and optic neuropathy has not been established, but this case may suggest peripheral precedes optic neuropathy. This aligns with the improvement of visual symptoms but not extremity numbness. Pain and paresthesia would require greater recovery time due to the prolonged drug induced mitochondrial damage to the nerves.

## 4 Conclusion

We present a case of linezolid toxic optic neuropathy. A unique feature to our case is a history of pentosan polysulfate use which was determined not clinically significant. Existing case reports suggest that many cases will have some residual deficit after recovery. Some resources recommend ophthalmic exam when treatment exceeds 3 months.^16^ The authors believe this is an acceptable recommendation as the average time to symptom onset was twelve months.

## Patient consent

Consent to publish the case report was not obtained. This report does not contain any personal information that could lead to the identification of the patient.

## Declaration of competing interest

The authors declare that they have no known competing financial interests or personal relationships that could have appeared to influence the work reported in this paper.
